# Comparison of Critical Illness Score in Patients Admitted to Intermediate Care Units of a Tertiary Care Hospital: A Comparative Cross-Sectional Study from Karachi, Pakistan

**DOI:** 10.2478/jccm-2024-0003

**Published:** 2024-01-30

**Authors:** Amber Sabeen Ahmed, Madiha Iqbal, Sher Muhammad Sethi, Sania Sabir, Aysha Almas

**Affiliations:** Aga Khan University Hospital, Karachi, Pakistan

**Keywords:** APACHE, critical illness, early warning score, simplified acute physiology score, SOFAS

## Abstract

**Introduction:**

Intermediate care units (IMCUs) serve as step-up units for emergency department patients and as step-down units for critically ill patients transferred from intensive care units. This study compares four critical illness scores for assessment of acutely ill patients and their accuracy in predicting mortality in patients admitted to IMCU.

**Methods:**

A comparative cross-sectional study on patients aged ≥18 admitted to IMCU of Aga Khan University Hospital from 2017 to 2019. All patients admitted to IMCU from the emergency room were included in the study. Patient’s record were reviewed for demographic data, physiological and laboratory parameters. Critical illness scores were calculated from these variables for each patient.

**Results:**

A total of 1192 patients were admitted to the IMCU, of which 923 (77.4%) medical records were finally analyzed. The mean (SD) age of participants was 62 years (± 16.5) and 469 (50.8%) were women. The overall hospital mortality rate of patients managed in IMCU was 6.4% (59/923 patients). The median scores of APACHE II, SOFA, SAPS II and MEWS were 16 (IQR 11–21), 4 (IQR 2–6), 36 (IQR 30–53) and 3 (IQR 2–4) points respectively. AUC for SAPS II was 0.763 (95% CI: 0.71–0.81), SOFA score was 0.735 (95% CI: 0.68–0.79) and MEWS score was 0.714 (95% CI: 0.66–0.77). The lowest ROC curve was 0.584 (95% CI: 0.52–0.64) for APACHE II.

**Conclusion:**

In conclusion, our study found that SAPS II, followed by SOFA and MEWS scores, provided better discrimination in stratifying critical illness in patients admitted to IMCU of a tertiary care hospital in Pakistan.

## Introduction

Critically ill patients often require admission in intensive care units (ICU) which are equipped with intensive patient monitoring and also requires 1:1 nurse to patient ratios.[1, 2]. In lower- and middle-income countries (LMIC) like Pakistan, ICU are often limited and many times patients are admitted to intermediate care units (IMCU)s or high dependency units (HDU) [3]. IMCU is defined as an independent unit which can accommodate group of patients who are sick and require vigorous monitoring. It can act as a “step-up” units for patients who are admitted from emergency department to these units instead of general wards and can act as “step-down” units for critically ill patients who were admitted in intensive care units [4, 5].

Most scoring systems are measures of the severity of diseases and are used to predict mortality in patients in ICU [6]. Deterioration of patients and subsequent need for ICU admission have led to a demand for risk stratification tools for early recognition of clinical deterioration [7]. Most research on prognostication of these acute medical conditions has been done in ICU and not in IMCU [8]. In LMIC like Pakistan, the ICU beds are limited and many semi critical to critical patients end up in IMCU/HDU [9]. Hence scoring systems for prognostication of such patients in IMCU is absolutely essential.

Each of these scoring systems uses a different combination of parameters to stratify the patients. Scoring systems play an essential role in improving clinical decisions, and identifying patients with unexpected outcomes. In previous studies, many of these scores have been confirmed to be clinically useful in predicting early deterioration in a patient’s clinical condition mostly in patients admitted to ICU [6].

Different scoring systems have been used to assess the severity and prognosis of the disease in critical care. Acute Physiology and Chronic Health Evaluation II (APACHE II), Sequential Organ Failure Assessment (SOFA), Modified Early Warning Score (MEWS) and Simplified Acute Physiology Score II (SAPS II) are common critical illness scores measured in patients admitted to ICU. There is no evidence to suggest that they reflect the severity of illness in patients admitted to the IMCU. The main aim of this study was to compare these four critical illness scores including APACHE II, SOFA, SAPS II and MEWS of acutely ill patients admitted to IMCU and compare the predictive accuracy of these four scoring systems in predicting mortality in these units.

## Method

This was a comparative cross-sectional study, carried out on patients aged 18 or above admitted to IMCU of medical ward of Aga Khan University Hospital (AKUH). The study was conducted after getting an exemption from the Ethical Review Committee of the institute (ERC Number: 4990-Med-ERC-17). The informed consent was waived by the ERC of our institute. The study duration was from 2017 to 2019.

AKUH is a Joint commission for International Accreditation (JCIA) certified 650 bedded tertiary care university hospital located in Karachi, Pakistan. At AKUH there is 25 bedded IMCU which is geographically distributed over 3 sites. IMCU is an open unit for acute medical patients who need continuous vitals monitoring along with continuous pulse oximetry and they can be offered noninvasive ventilation also. The registered nurse (RN) to patient ratio is 1:5 in the IMCU and the RN also has support of a nursing assistant all the time for general care of the patient.

This study included all adult patients aged 18 and above with acute medical illnesses who were admitted directly from emergency rooms to internal medicine intermediate care units (IMCU). Patients who were admitted to ICU and general wards and later shifted to IMCU were excluded. Patients who had advanced care directive with do not resuscitate (DNR) order, and patients who were transferred from other services of the hospital to Internal Medicine services were also excluded. Patients who left against medical advice were also excluded from this study.

Data was collected from both patient’s electronic medical records and review of patient’s files. The investigators collected the data and personal identifier of the patients were not recorded to keep the data anonymous. Demographic variables like age, admitting diagnosis, comorbidities, in-hospital management, duration of hospitalization, and condition on discharge were recorded. The patient’s files were reviewed for physiological parameters i.e. blood pressure, heart rate, respiratory rate, urine output, and, laboratory parameters. APACHE II, SOFA, MEWS, and SAPS were calculated from these variables for each patient (Appendix). APACHE II is based on 12 physiological measurements, age and previous health status and is designed to provide a measurement of the severity of the disease [10]. The SOFA score identifies the number and severity of failed organs and provides prognostic information on mortality rates [11]. MEWS is a simple medical score that evaluates vital signs and is useful for risk assessment about the severity of illness [12]. While SAPS score comprises 17 components, including 12 physiological variables, age, admission type and underlying disease. It provides an estimation of mortality rates for different medical conditions [13]. Primary outcome of the study was in hospital mortality was taken as outcome variable.

### Statistical analysis

Statistical analysis was performed through SPSS version-22. Mean and standard deviation was used for quantitative variable and frequency and percentage for categorical variable. Median score and interquartile range for each severity score was calculated for the scores due to their skewed distribution. Comparison between survivors and non survivors were made using a 2 sample T-test. Logistic regression analysis was also used to assess the relationship between different patient related variables and in hospital mortality.

Accuracy was checked by assessing how well predicted mortality by APACHE score matched the actual mortality. Discrimination of each score was assessed. If a model assigns a higher risk of death to patients who die and lower risk to patients who survive, it is said to have good discriminative properties. Discrimination is characterized by the area under receiver operating curve (AUROC) with an area of 1 indicating perfect discrimination and area of 0.5 indicating no discrimination. Comparison of AUROC of different scores were done. Finally standardized mortality ratio (SMR=observed deaths/predicted deaths) was calculated for all models. The statistical methods were verified, assuming a significance level of p <0.05.

## Results

A total of 1192 patients were admitted in the IMCU during the study period out of which 923 medical records were finally analyzed (136 (11%) patient’s medical records data were not available and 79 (7.8%) patients had advanced care directive with do not resuscitate order, and 54 (4%) patients were transferred from other services of the hospital to Internal Medicine IMCU). Mean (SD) age of participants were 62 (± 16.5) years and 469 (50.8%) were women. The most common acute medical illness admitted in IMCU were acute kidney injury; 373 (40%), followed by pneumonia; 265 (27.7%), and urinary tract infections; 201 (21.8%). The overall hospital mortality rate of patients managed in IMCU was 6.4% (59/923 patients).

### Comparison of survivor and non-survivor group

The age group 60–80 had the largest number of patients (55.62%) in survivor group while the age group 40–60 forms the largest cohort in non-survivors (44.23%). Hypertension was the most frequent comorbid in both of the groups with 70% of patients in the survivor group and 66% in non-survivor group having this condition. Diabetes was the next most frequent comorbid condition present in more than half of the patients in both group (54.9%in survivor group and 57.6% in the non-survivor group).

In our study, the overall mortality was 59 patients (6.4%). The acute medical conditions that were significantly different between survivor and non-survivor were; sepsis and septic shock {107(12.3%) versus 23(38.9%), p value <0.001}, pneumonia {235(27.1%) versus 30(50.8%), p value 0.009}, and myocardial infarction / pulmonary edema {62(7.17%) versus 9 (15.25%), p value 0.038}.

When we analyzed individual diseases we found septic shock to be having the highest mortality rates. There were 130 patients in the study cohort with working diagnosis of septic shock. Out of these 107 patients were survivors and 23 patients were non-survivors, with mortality rate of 17.69% for the disease. Two hundred and sixty five patients were admitted with diagnosis of Pneumonia. Majority of them survived (235/923) and mortality related to the disease was found to be only 11.2%. Myocardial infarction (MI) incidence was comparatively lower than other disease conditions. There were 71 patients in the study cohort who had acute MI. Out of them 62 patients survived the disease and the mortality associated with this condition was 12.67% (9/71).

The length of hospital stay was nearly equal in both group and was found to be 5.9 ± 4.7 (mean ± SD) days.

The demographic, comorbid and top 10 acute conditions and outcome overall, survivors and non survivors are shown in Table 1.

**Table 1.** Demographic, comorbid and top 10 acute conditions and outcome overall, survivors and non survivors of the patients admitted to Intermediate care medical units (N=923)

**Characteristic**	**N=923 (%)**	**Survivors N=864/923(%)**	**Non-survivors N=59/923 (%)**	**P-value**
Age, Mean (SD), years	62	61.2	63.6	0.86

Age Groups				
Up to 20 years	1.49%	1.57%	0	
20 – 40 years	10.46%	10.39%	7.69%	
40 – 60 years	34.16%	33.96%	44.23%	
60 – 80 years	46.06%	55.62%	34.61%	
80+ years	11.86%	11.62%	13.46%	

Gender				
Male (%)	454 (49.2%)	421 (48.7%)	33 (55.9%)	0.284
Female (%)	469 (50.8%)	443 (51.3%)	26 (44.1%)	

Top 10 common diagnosis				
Sepsis, septic shock n, (%)	130(13.1%)	107(12.3%)	23(38.9%)	0.000
Pneumonia n, (%)	265(27.7 %)	235(27.1%)	30(50.8%)	0.009
UTI n, (%)	201(21.8%)	193(22.33%)	8(13.55%)	0.142
AKI n, (%)	373(40%)	350(40.5%)	23(38.9%)	0.817
MI and Pulmonary Edema n, (%)	71(7.7%)	62(7.17%)	9(15.25%)	0.038
Stroke n, (%)	28(3.0%)	24(2.77%)	4(6.77%)	0.097
Atrial Fibrillation n, (%)	28(3.0 %)	24(2.77%)	4(6.77%)	0.083
Electrolyte Imbalance n, (%)	170(18.4%)	162(18.75%)	8(13.55%)	0.387
Exacerbation of Obstructive Airway Disease n, (%)	59(6.4 %)	53(6.13%)	6(10.16%)	0.220
Solid Tumors n, (%)	26(2.8%)	23(2.66%)	3(5.08%)	0.403

Comorbid n, (%)				
Diabetes	510 (55.3%)	472(54.62%)	34(57.62%)	0.650
Hypertension	648 (70.2%)	609(70.48%)	39(66.10%)	0.470
Ischemic Heart Disease	307(33.3%)	230(26.62%)	21(35.55%)	0.134
Chronic Kidney Disease	474 (51%)	216(25.00%)	24(40.67%)	
Chronic Liver Disease	66 (7.2%)	56(6.48%)	10(16.94%)	0.003
Thyroid Disorders	37 (4.0%)	36(4.16%)	01(1.69%)	0.349
Malignancy	26 (2.8%)	24(2.77%)	2(3.38%)	0.783
HIV/AIDS	7 (0.7%)	6(0.69%)	01(1.69%)	0.391

Length of stay (mean ± SD)	5.9 ± 4.7			

Mortality rate n, (%)	59 (6.4%)			

SD: standard deviation; UTI: urinary tract infection; AKI: acute kidney injury; MI: myocardial infarction; HIV/AIDS: human immunodeficiency virus/acquired immunodeficiency syndrome

### Critical illness scores

The median scores of APACHE II, SOFA, SAPS II and MEWS were 16 (IQR 11–21), 4 (IQR 2–6), 36 (IQR 30–53) and 3 (IQR 2–4) points respectively.

Comparison of the scores between survivors and non-survivors along with area under receiver operator curve (AUROC), sensitivity and specificity of various scores is shown in Table 2.

**Table 2.** Comparison of critical illness scores overall, among survivors and non-survivors in patients admitted to intermediate care units

**Critical Illness scales Median (interquartile range)**	**Overall (n=923)**	**Survived (n=864)**	**Not survived (n=59)**	**P-value**
APACHE II	16(11–21)	16(11–21)	20(13–25)	0.002
SOFA	4(2–6)	4(2–5)	7(4–10)	0.000
SAPS II	36(30–53)	35(30–42)	44(38–53)	0.000
MEWS	3(2–4)	3(2–4)	5(3–6)	0.000

APACHE: Acute Physiology and Chronic Health Evaluation; SOFA: Sequential Organ Failure Assessment; SAPS: Simplified Acute Physiology Score; MEWS: Modified Early Warning Score

**Fig. 1.**
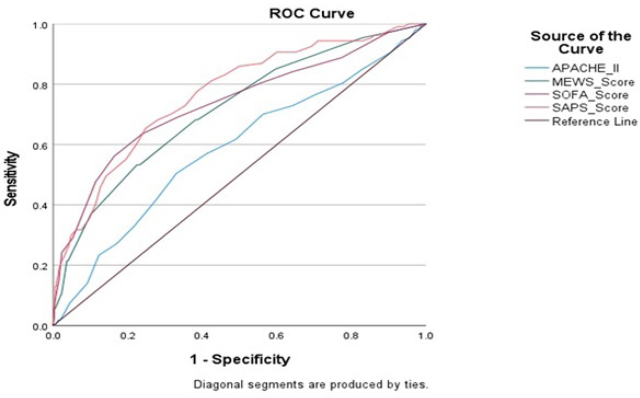
ROC Curve showing four different critical illness scores in patients admitted to IMCU

Area under the curve (AUC) of the four scoring system for predicting mortality were obtained from their receiver operating curves (ROC). The AUC for SAPS II was 0.763 (95% CI: 0.71–0.81) which was highest among all the scores studied. The AUC for SOFA score was found to be 0.735 (95% CI: 0.68–0.79) and for MEWS score it was 0.714 (95% CI: 0.66–0.77). The lowest ROC curve among all the scores studied in our study was 0.584 (95% confidence interval [CI].: 0.52–0.64) for APACHE II. Figure 1 shows a plotted graph that compares ROC of all the four scores.

Cutoffs were calculated based on the sensitivity and specificity achieved form ROC curves. The cutoffs for the four scoring system were as follows: SOFA score ≥6 (sensitivity 64% and specificity 76%, SAPS II ≥39 (sensitivity= 78% and specificity= 62%), MEWS ≥3 (sensitivity= 85% and specificity= 40%) and APACHE II ≥12 (sensitivity= 70% and specificity= 76.0%).

We also calculated the standardized mortality rate (SMR) based on mortality prediction by different scores. SAPS II has substantially overestimated mortality with SMR = 0.35. APACHE-II score also substantially overestimated mortality (SMR=0.256). SOFA score moderately overestimated mortality with SMR=0.64 and MEWS also showed nearly similar mortality prediction with SMR=0.51. (Table 3)

**Table 3.** The area under the characteristic curve, sensitivity, and specificity data of critical illness scores in patients admitted to intermediate care units

**Scores**	**Cutoff values**	**AUC (95%CI)**	**P value**	**Sensitivity %**	**Specificity %**	**SMR (95%CI)**
Acute Physiology and Chronic Health Evaluation II	≥ 12	0.584 (0.52–0.64)	0.004	70	76	0.25 (0.32–0.19)
Sequential Organ Failure Assessment	≥ 6	0.735 (0.68–0.79)	0.000	64	76	0.64 (0.80–0.48)
Simplified Acute Physiology Score II	≥ 39	0.763 (0.71–0.81)	0.000	78	62	0.35 (0.45–0.26)
Modified Early Warning Score	≥ 3	0.714 (0.66–0.77)	0.000	85	40	0.51 (0.64–0.38)

## Discussion

In this study, we found SAPS II as performing better in predicting mortality in IMCU patients among all the four scores studied. SAPS II was found to have an AUROC of 0.76% (95% confidence interval (95%CI: 0.71 to 0.81) which is the highest AUC among the evaluated scores, indicating good predictive accuracy. It showed higher sensitivity (78%) but lower specificity (62%). SOFA score had AUROC of 0.74(95%CI: 0.68–0.79) indicating comparable predictive accuracy to SAPS II. However, it exhibited a lower sensitivity (64%) and increased specificity (76%) compared to SAPS II. MEWS score with AUROC of 0.71(95%CI: 0.66–0.77) showed moderate predictive accuracy. It had the highest sensitivity (85%) among all the scores, indicating its proficiency in identifying critically ill patients. However, it had lower specificity (40%), suggesting a higher rate of false positives. The lower specificity could lead to unnecessary interventions or investigations in patients who are not critically ill. APACHE II which is known validated scoring system for mortality prediction in ICU patients was found to have the lowest AUC of 0.58 (95%CI: 0.52–0.64) of all the scoring systems. suggesting moderate accuracy in predicting patient outcomes. It demonstrated relatively balanced sensitivity (70%) and specificity (76%), indicating its ability to identify both critically ill patients and non-critically ill patients.

The discriminatory performance of SAPS II in our IMCU population is comparable to other studies done in French, Spanish and North American intermediate care units [14,15,16]. All of these studies done in different parts of the world at different point in time, also showed SAPS II as exhibiting good performance characteristics among different severity scores. In the study from the United States, Brusc et al reported SAPS II as having the best discrimination power among all the scores studied in an intermediate care unit of an academic medical centre in United States. They report AUC of SAPS II in their cohort of 628 patients of around 0.80 (95%CI: 0.74 – 0.87) [14]. The French study which was done in a close IMCU, which were located in close proximity to ICU, in a population of 433 patients also showed SAPS II to have the best discrimination and calibration with AUC of around 0.85 [16]. In another study done in Spain by Lucena et al. on 607 patients admitted to a close bed IMCU, again SAPS II score showed AUC of 0.85 with good calibration and SMR of 0.87 [15]. Our study also demonstrate a similar result showing AUC for SAPS II of around 0.76 (95%CI: 0.67 – 0.80) and was much better in predicting severity of the illness. SAPS II uses admission criteria in scoring calculation along with acute physiological parameters. The use of admission criteria variable might be important in intermediate care unit patients’ risk estimation compared to ICU patients and might be the reason behind SAPS II‘s superior performance beside other. It also has a higher sensitivity (78%) compared to both APACHE II and SOFA, indicating its proficiency in identifying critically ill patients. However, its specificity is lower at 62%, which means it may result in more false positives. In contexts like IMCU setting where it is more important to correctly identify critically ill patients while accepting a higher false positive rate, SAPS II could be a suitable choice.

The observed mortality rate in our study was 6.4% (59/923 patients). Predicted mortality rate of all the scores in our study, overestimated the actual mortality rate in our patient population. The standardized mortality rate (SMR) which is the ratio of observed death to expected death was found low for all four of the scores studied. Even SAPS II which showed comparable discrimination properties (AUROC = 0.763) to the studies done in Spain, France and USA markedly overestimated the mortality rate (SMR =0.35) [10,11,12]. SOFA score moderately overestimated mortality (SMR=0.64) and MEWS also showed nearly similar mortality prediction (SMR=0.51). APACHE II one of the most widely used severity score has also substantially overestimated mortality (SMR=0.256).

If we look at both discrimination and SMR of all the scores in our study, we found SOFA score to be perform better than other scores studied with AUROC comparable to SAPS II in our study (0.73 of SOFA versus 0.76 of SAPS II) and also SMR of 0.64.

SOFA is a validated score for septic population and also for ICU patients. Innocenti et al. in a study on HDU or IMCU patients (N=3,311) admitted in Italian hospital, demonstrated a good discriminatory ability of SOFA score for HDU mortality (AUROC of 0.80 with Confidence interval of 0.70–0.91, compared with MEWS, SAPS, and APACHE score) [17].

In another study from Germany, done on 13,780 surgical patients who were either treated in IMCU, ICU or both of the units, between Ist.January’2012 and 30th September’2018 by Christian Koch et al reported SOFA score as having good mortality prediction for mixed ICU/IMCU population. The AUROC of SOFA score for mix ICU/IMCU cohort was 0.73 [0.70–0.77]. Although for specific IMCU cohort, q-SOFA score performed best in that study with AUROC of 0.82 [0.79–0.84]. compared to SOFA which had AUROC of 0.52 [0.51–0.53] [18]. SOFA score in all these studies and also in our study was taken at the time of admission whereas SAPS II and APACHE II scores were calculated from the most aberrant data in the 24 hours following admission. Collecting data at time of admission probably makes SOFA score more efficient and useful tool and remove biases related to management in term of mortality prediction. However, its sensitivity is lower at 64%, indicating it may miss some critically ill patients. On the other hand, its specificity is 76%. In contexts where it is more crucial to avoid false negatives (missing critically ill patients in IMCU requiring ICU transfer), the lower sensitivity may be a concern.

In our study APACHE II which is one of the most validated tool for ICU patients, did not perform well in terms of both discrimination and mortality prediction for our IMCU patient population [19,20,21]. This in contrast to the study done by Brusca et al. on IMCU patients which showed AUROC for APACHEII as 0.76 (95%CI: 0.70–0.83) which showed good discrimination [14]. Our study findings for APACHE II are similar to the study done on IMCU patients by Jahn et al. at a German University hospital [22]. The patient population though was entirely made of cirrhotic patients and therefore not similar to our mix disease patient population. That study also showed SAPS II as the most superior score. It could be that APACHE II is designed for ICU patients and therefore probably not suitable for comparatively stable IMCU patients. The reason that APACHE could not have good discriminative power in these patients admitted in IMCU could be that APACHE is a very detailed and includes blood gas analysis linked to invasive ventilation, which adds to the totals score, while SAPS, SOFA and MEWS do not have the blood gas component (mandatory for invasive ventilation) and therefore have better discriminative power for the non-ventilated IMCU patients.

While it may not have the highest AUC among the evaluated scores, its well-balanced sensitivity and specificity could be beneficial in situations like IMCU where it is essential to capture both true positive and true negative cases with similar importance.

In our study the overall mortality rate was low because it was conducted at an IMCU which has lower mortality in comparison to ICU throughout the world. The low observed mortality rates to predict severity scores could be attributed to good care quality and organization of our intermediate care unit. The intermediate care unit at AKUH have guidelines and protocols for common acute conditions which are based on international guidelines and similar to the ones used in our ICU. Our study population was relatively young (mean age 62 years old) which could have influenced the observed mortality rate. Admitting diagnosis of pneumonia, sepsis and septic shock or acute MI were strong mortality predictors in our study but were present in a minority of patients admitted to IMCU. In our study population twenty seven percent of patients had admitting diagnosis of pneumonia. Sepsis/septic shock was present in only 11 percent of the patient population and acute MI was reason for admission in only 7% of our patient population.

The strength of this study is that it’s one of the first in intermediate care unit comparing critical illness scores of patients admitted with a large sample size. The study also demonstrates that IMCU serve as a bridging unit for critically ill patients in LMIC like Pakistan as the number of ICU beds are often limited. There are few limitations to our study. Our study was a single centered study which limits generalizability of the study. We only took patients admitted in medical IMCUs and not cardiac or surgical IMCUs. In our study we did not look at the financial benefits of offering treatment in IMCU compared to ICU as in an economically challenged situation, the cost effectiveness of the care has acquired significant importance.

## Conclusion

When considering sensitivity, specificity, and AUC, the Simplified Acute Physiology Score II (SAPS II) appeared to be the most balanced and effective critical illness score. It had the highest AUC, indicating better overall predictive accuracy, and a relatively high sensitivity (78%) to identify critically ill patients effectively. While its specificity (62%) was not as high as some other scores, it remained within a reasonable range. However, clinicians and researchers should select a suitable score based on the clinical context and the desired balance between correctly identifying critically ill patients and minimizing false alarms.

We need further studies on the topic to find an ideal critical illness score for IMCU patients.

## Appendix Score Calculation and Interpretation

**Table:**

**Score**	**Calculation**	**Interpretation**	
SOFA	PiO_2_/FiO_2_: (mmHg) <400 = 1 point, <300 = 2 points, <200 = 3 points, <100 = 4 points	Score	Mortality
	Serum Creatinine: (mg/dl) 1.2–1.9 = 1 point, 2.0–3.4 = 2 points, 3.5–4.9 = 3 points, ≥5 = 4 points	0– 6 7 – 9 10 – 12 13 – 14 15 15– 24	<10% 15–20% 40–50% 50–60% >80% >90%
	Platelets: (× 10^3^/uL) <150 = 1 point, <100 = 2 points, <50 = 3 points, <20 = 4 points		
	GCS: (out of 15) 13–14 = 1 point, 10–12 = 2 points, 6–9 = 3 points, <6 = 4 points		
	MAP/Use of Vasopressor: MAP < 70 mm/Hg = 1 point dopamine ≤ 5 μg/kg/min or dobutamine (any dose) = 2 point dopamine > 5 μg/kg/min OR epinephrine ≤ 0.1 μg/kg/min OR norepinephrine ≤ 0.1 μg/kg/min = 3 points dopamine > 15 μg/kg/min OR epinephrine > 0.1 μg/kg/min OR norepinephrine > 0.1 μg/kg/min = 4 points		
	Bilirubin: (mg/dl) 1.2– 1.9 = 1 point, 2.0– 5.9 = 2 points, 6.0 – 11.9 = 3points, >12 = 4 points		

MEWS	RR: (breath/min) ≤8 = 2 points, 15–20 = 1 point, 21–29 = 2 points, ≥30 = 3 points	Score	Plan
	HR: (beats/min) <40 = 2 points, 40–50 = 1 point, 101–110 = 1 point 111–129 = 2 points, >129 = 3points	1–2	2 hour observation
	Systolic blood pressure: (mmHg) ≤70 = 3 points, 71–80 = 2 points, 81–100 = 1 point, >200 = 2 points	3	1–2 hour observation
	Temperature: (°C) ≤34 = 3 points, 34.1–35 = 2 points, 35.1–36 = 1 point, 38–38.5 = 1 point, ≥38.6 = 2 points	≥4	1/2hr observe & inform doctor
	CNS: Unresponsive = 3 points, Respond to pain = 2 points, Respond to voice/New agitation/Confusion = 1 point		
	Urine Output: (ml in 24hr) <480 = 2 points, 480–714 = 1 point, >4800 = 1 point		

APACHE II	Age: (<44 years = 0 point) 45–54 = 2 points, 55–64 = 3 points, 65–74 = 5 points, >74 = 6 points	Score	Mortality Rate
	Temperature: (Normal: 36– 38.4°C) ≥ 41 / ≤ 29.9 = 4 points, 39–40.9 / 30–31.9 = 3 points,- / 32–33.9 = 2 points, 38.5–38.9 / 34–35.9 = 1 point	0–4	4%
	Mean Arterial Pressure (Normal: 70–109 mmHg) ≥160 / ≤ 49 = 4 points, 130–159 /- = 3 points, 110–129 / 50–69 = 2 points	5–9	8%
	Heart Rate (Normal: 70–109 bpm) ≥180 / ≤ 39 = 4 points, 140–179 / 40–54 = 3 points, 110–139 / 55–69 = 2 points	10–14	15%
	Respiratory Rate (Normal: 12–24 breath/min) ≥50 / ≤ 5 = 4 points, 35–49 /- = 3 points,- / 6–9 = 2 points, 25–34 / 10–11 = 1 point	15–19	25%
	Oxygenation (mmHg) If FiO_2_ ≥ 0.5, use A-a O_2_: ≥500 = 4 points, 350–499 = 3 points, 200–349 = 2 points, <200 = 0 point If FiO_2_ < 0.5, use P_A_O2: <55 = 4 points, 55–60 = 3 points, 61–70 = 1 points, >70 = 0 point	20–24	40%
	Arterial pH (Normal: 7.33–7.49) ≥ 7.7 / ≤ 7.15 = 4 points, 7.6.7.69 / 7.15–7.24 = 3 points,- / 7.25–7.32 = 2 points, 7.5–7.59 /- = 1 point	25–29	55%
	Serum HCO_3_ (N: 22–31.9 mmol/L) ‘if ABGs unavailable’ ≥ 52 / < 15 = 4 points, 41–51.9 / 15–17.9 = 3 points,- / 18–21.9 = 2 points, 32–40.9 /- = 1 point	30–34	75%
	Serum Na (Normal: 130–149 mmol/L) ≥ 180 / ≤ 110 = 4 points, 160–179 / 111–119 = 3 points, 155–159 / 120–129 = 2 points, 150–154 /- = 1 point	>34	85%
	Serum K (Normal: 3.5–5.4 mmol/L) ≥ 7 / ≤ 2.5 = 4 points, 6–6.9 /- = 3 points,- / 2.5–2.9 = 2 points, 5.5–5.9 / 3–3.4 = 1 point		
	Serum Cr (Normal: 0.6–1.4 mg/dL) Score × 2 (if AKI) ≥ 3.5 /- = 4 points, 2–3.4 /- = 3 points, 1.5–1.9 / <0.6 = 2 points		
	Hematocrit (Normal: 30–45.9%) ≥ 60 / < 20 = 4 points, 50–59.9 / 20–29.9 = 2 points, 46–49.9/- = 1 point		
	WBCs (Normal: 3–14.9 ×10^3^/uL) ≥ 40 / < 1 = 4 points, 20–39.9 / 1–2.9 = 2 points, 15–19.9 /- = 1 point		
	GCS = Score: 15 – actual GCS		
	Chronic Health Status: 2 points = elective postoperative patient with immunocompromise or history of severe organ insufficiency 5 points = non-operative patient or emergency postoperative patient with immunocompromise or severe organ insufficiency		

SAPS II	Temperature (°C) <39 = 0 point, ≥39 = 3 points	Score	Mortality Rate
	Systolic Blood Pressure (mmHg) <70 = 13 points, 70–99 = 5 points, 100–199 = 0 point, ≥200 = 2 points	29	10%
	Heart Rate (bpm) ≥ 160 = 7 points, 120–159 = 4 points, 70–119 = 0 point, 40–69 = 2 points, <40 = 11 points	40	25%
	PaO_2_/FiO_2_ (mmHg) = only if on VENT or CPAP <100 = 11 points, 100–199 = 9 points, ≥200 = 7 points	52	50%
	Urine Output (liter/day) <0.5 = 11 points, 0.5–0.99 = 4 points, ≥ 1 = 0 point	64	75%
	Urea (g/L) <0.6 = 0 point, 0.6–1.7 = 6 points, >1.88 = 10 points	77	90%
	Serum HCO_3_ (mmol/L) <15 = 6 points, 15–19 = 3 points, >20 = 0 point		
	Serum Na (mmol/L) <125 = 5 point, 125–144 = 0 point, ≥145 = 1 point		
	Serum K (mmol/L) < 3 = 3 points, 3–4.9 = 0 point, ≥ 5 = 2 points		
	WBCs (×10^3^/uL) < 1 = 12 points, 1–19.9 = 0 point, ≥ 20 = 2 points		
	Bilirubin (mg/dL) < 40 = 0 point, 40–59.9 = 4 points, ≥ 60 = 9 points		
	GCS (out of 15) < 6 = 26 points, 6–8 = 13 points, 9–10 = 7 points, 11–13 = 5 points, 14–15 = 0 point		
	Age (years) <40 = 0 point, 40–59 = 7 points, 60–69 = 12 points, 70–74 = 15 points, 75–79 = 16 points, >80 = 18 points		
	Chronic disease Metastatic cancer = 9 points, Hematological malignancy = 10 points, AIDS = 17 points		
	Type of Admission Elective/Surgery = 0 point, Medical = 6 points, Emergency Surgery = 8 points		

## References

[1] Sharma SK, Rani R (2020). Nurse-to-patient ratio and nurse staffing norms for hospitals in India: A critical analysis of national benchmarks. J Family Med Prim Care.

[2] Smith G, Nielsen M (1999). ABC of intensive care. Criteria for admission. BMJ.

[3] Khan MA, Shahbaz H, Noorali AA, Ehsan AN, Zaki M, Asghar F (2022). Disparities in adult critical care resources across Pakistan: findings from a national survey and assessment using a novel scoring system. Crit Care.

[4] Plate JDJ, Leenen LPH, Houwert M, Hietbrink F (2017). Utilisation of Intermediate Care Units: A Systematic Review. Crit Care Res Pract.

[5] Sjoding MW, Valley TS, Prescott HC, Wunsch H, Iwashyna TJ, Cooke CR (2016). Rising Billing for Intermediate Intensive Care among Hospitalized Medicare Beneficiaries between 1996 and 2010. Am J Respir Crit Care Med.

[6] Vincent JL, de Mendonca A, Cantraine F, Moreno R, Takala J, Suter PM (1998). Use of the SOFA score to assess the incidence of organ dysfunction/failure in intensive care units: results of a multicenter, prospective study. Working group on “sepsis-related problems” of the European Society of Intensive Care Medicine. Crit Care Med.

[7] Chawda MN, Hildebrand F, Pape HC, Giannoudis PV (2004). Predicting outcome after multiple trauma: which scoring system?. Injury.

[8] Keegan MT, Gajic O, Afessa B (2011). Severity of illness scoring systems in the intensive care unit. Crit Care Med.

[9] Amber Sabeen Ahmed EH, Haleem Sohail, Ahmed Naila, Latif Asad (2021). Epidemiology of sepsis, based on ICD-9 coding, a tertiary care experience from Pakistan. Trends in Anaesthesia and Critical Care.

[10] Knaus WA, Draper EA, Wagner DP, Zimmerman JE (1985). APACHE II: a severity of disease classification system. Crit Care Med.

[11] Jones AE, Trzeciak S, Kline JA (2009). The Sequential Organ Failure Assessment score for predicting outcome in patients with severe sepsis and evidence of hypoperfusion at the time of emergency department presentation. Crit Care Med.

[12] Gardner-Thorpe J, Love N, Wrightson J, Walsh S, Keeling N (2006). The value of Modified Early Warning Score (MEWS) in surgical in-patients: a prospective observational study. Ann R Coll Surg Engl.

[13] Le Gall JR, Lemeshow S, Saulnier F (1993). A new Simplified Acute Physiology Score (SAPS II) based on a European/North American multicenter study. JAMA.

[14] Brusca RM, Simpson CE, Sahetya SK, Noorain Z, Tanykonda V, Stephens RS (2020). Performance of Critical Care Outcome Prediction Models in an Intermediate Care Unit. J Intensive Care Med.

[15] Lucena JF, Alegre F, Rodil R, Landecho MF, Garcia-Mouriz A, Marques M (2012). Results of a retrospective observational study of intermediate care staffed by hospitalists: impact on mortality, co-management, and teaching. J Hosp Med.

[16] Auriant I, Vinatier I, Thaler F, Tourneur M, Loirat P (1998). Simplified acute physiology score II for measuring severity of illness in intermediate care units. Crit Care Med.

[17] Innocenti F, Bianchi S, Guerrini E, Vicidomini S, Conti A, Zanobetti M (2014). Prognostic scores for early stratification of septic patients admitted to an emergency department-high dependency unit. Eur J Emerg Med.

[18] Koch C, Edinger F, Fischer T, Brenck F, Hecker A, Katzer C (2020). Comparison of qSOFA score, SOFA score, and SIRS criteria for the prediction of infection and mortality among surgical intermediate and intensive care patients. World J Emerg Surg.

[19] Sungono V, Hariyanto H, Soesilo TEB, Adisasmita AC, Syarif S, Lukito AA (2022). Cohort study of the APACHE II score and mortality for different types of intensive care unit patients. Postgrad Med J.

[20] Castella X, Artigas A, Bion J, Kari A (1995). A comparison of severity of illness scoring systems for intensive care unit patients: results of a multicenter, multinational study. The European/North American Severity Study Group. Crit Care Med.

[21] Markgraf R, Deutschinoff G, Pientka L, Scholten T (2000). Comparison of acute physiology and chronic health evaluations II and III and simplified acute physiology score II: a prospective cohort study evaluating these methods to predict outcome in a German interdisciplinary intensive care unit. Crit Care Med.

[22] Jahn M, Raschidi L, Ozcurumez MK, Arzideh F, Korth J, Kribben A (2022). Comparison of Mortality Prediction Scores in Intermediate-Care Patients with Liver Cirrhosis at a German university transplant Center; a prospective study. Dig Dis.

